# A Patient-Centered Framework for Evaluating Digital Maturity of Health Services: A Systematic Review

**DOI:** 10.2196/jmir.5047

**Published:** 2016-04-14

**Authors:** Kelsey Flott, Ryan Callahan, Ara Darzi, Erik Mayer

**Affiliations:** ^1^ Centre for Health Policy Institute of Global Health Innovation Imperial College London London United Kingdom

**Keywords:** digital maturity, evaluation, health information exchange, patient-centered care

## Abstract

**Background:**

Digital maturity is the extent to which digital technologies are used as enablers to deliver a high-quality health service. Extensive literature exists about how to assess the components of digital maturity, but it has not been used to design a comprehensive framework for evaluation. Consequently, the measurement systems that do exist are limited to evaluating digital programs within one service or care setting, meaning that digital maturity evaluation is not accounting for the needs of patients across their care pathways.

**Objective:**

The objective of our study was to identify the best methods and metrics for evaluating digital maturity and to create a novel, evidence-based tool for evaluating digital maturity across patient care pathways.

**Methods:**

We systematically reviewed the literature to find the best methods and metrics for evaluating digital maturity. We searched the PubMed database for all papers relevant to digital maturity evaluation. Papers were selected if they provided insight into how to appraise digital systems within the health service and if they indicated the factors that constitute or facilitate digital maturity. Papers were analyzed to identify methodology for evaluating digital maturity and indicators of digitally mature systems. We then used the resulting information about methodology to design an evaluation framework. Following that, the indicators of digital maturity were extracted and grouped into increasing levels of maturity and operationalized as metrics within the evaluation framework.

**Results:**

We identified 28 papers as relevant to evaluating digital maturity, from which we derived 5 themes. The first theme concerned general evaluation methodology for constructing the framework (7 papers). The following 4 themes were the increasing levels of digital maturity: resources and ability (6 papers), usage (7 papers), interoperability (3 papers), and impact (5 papers). The framework includes metrics for each of these levels at each stage of the typical patient care pathway.

**Conclusions:**

The framework uses a patient-centric model that departs from traditional service-specific measurements and allows for novel insights into how digital programs benefit patients across the health system.

**Trial Registration:**

N/A

## Introduction

Digital technologies are transforming health services by providing new mechanisms for accessing personal medical records, submitting incident reports, and communicating across care settings. In England, the government has placed the role of these technologies high on the agenda by setting a 3-year target for a fully paperless National Health Service (NHS). The *NHS Five Year Forward View*, which sets the strategic direction for the health service in England, focuses heavily on improving NHS digital technology with the aim of integrating all electronic medical records (EMRs) [1]. Furthermore, the continuing ambition of integrating health and social care relies on connected information technology (IT) systems. Amid this political climate and the influx of digital technologies, the potential for improvement is vast and certainly not limited to service operations; there is also scope to significantly improve the patient experience. There is an opportunity to depart from traditional service arrangements, overcome geographical boundaries, and even reconfigure services around the patients and their needs by harnessing digital technologies. However, these are not automatic byproducts of augmented IT programs. Like any care intervention, digital technologies need to be rigorously evaluated and monitored to ensure they operate in the way they are intended and cultivate a better experience across patient pathways. To conduct constructive appraisals, an evaluation framework is needed to make sure each factor that influences a digital system’s success is captured.

Digital maturity—the extent to which digital technologies are used as enablers to deliver a high-quality health service—is an emerging concept across developed health care systems, and there is no established measurement that accounts for all of its intricacies [2]. The adoption of digital solutions for EMRs across care services in Canada (facilitated by the Canada Health Infoway) provides existing examples of digital systems that have achieved a high level of maturity. However, the discourse surrounding digital maturity is dominated by ideas for its potential to improve services in the future. For instance, digital maturity is extensively cited as an aspirational goal necessary to join IT systems across care settings in order to effectively integrate health and social care services [3].

Example frameworks for evaluation from the health sector often focus on the operational benefits that individual services receive from digital technologies [4-6]. However, digital maturity is not about the success of one technological system and the benefits to one service’s particular stakeholders; rather, it is the advancement of the entire health service. To improve patient experience across care pathways, digital maturity must be measured in a way that addresses all the intersections it has across care settings and must be conceptualized in a way that dissolves the entrenched, service-specific standard for how digital systems are assessed.

The aim of our study was to propose a novel framework for evaluating digital maturity based on these principles. We summarized the existing evidence about how best to evaluate digital maturity and its component parts, and the merits of current digital maturity evaluations. Moreover, we synthesized evidence around what should be reflected in evaluations and developed a new framework capable of measuring digital maturity across the patient pathway and presenting it as a patient-centric, sectorwide achievement.

### Theory of Digital Maturity

The concept of digital maturity originated in the field of public service improvement. As more government services became IT enabled, they did so in siloes, meaning a single user (the citizen) would have to attempt to access information or obtain services from several different departments. Although the term digital maturity was not used at the time, the framework proposed by Layne and Lee in 2001 had 4 stages of integration to bring about citizen-centric e-government [7]. Their framework demonstrates that at its most mature level e-government represents the advancements and interworking of an entire field for the citizen; in health care this would be translated into the advancement of an entire field respectful of the way patients experience care.

Digital maturity builds on existing evidence about digital literacy. In healthcare this can primarily be captured in the idea of eHealth literacy or the ability of people to use information and communications technologies to improve or enable health care [8]. For health systems to support advancement of digital maturity, staff must be digitally literate and help patients improve their digital literacy. It is important to note, however, that while digital literacy can facilitate digital maturity, digital maturity should also be responsive to the whole patient population and account for their needs regardless of their digital literacy.

Gottschalk [9] and colleagues’ research moved beyond the concept of usability and linked the concept of maturity to greater interoperability. Their research posited a trajectory for how the content of interoperability tends to evolve—or mature—over time from the simplest level to more advanced, more integrated levels organized around the citizen. Because of the shift to organize services around the citizen, organizations would need to be increasingly interoperable, not just in terms of technical issues, but also in realizing benefits and setting goals. This is also mirrored in the health service as it becomes more integrated and individual services are required to communicate effectively across the patient pathway.

According to this research, digital maturity encompasses not only the resources and ability to use a system, but also how interoperable it is with other systems and ultimately its impact on the public. To understand how these aspects of digital maturity can be measured in the health service, a substantial body of literature provides guidance on how to build an evaluation framework for an information system.

## Methods

### Search Strategy

We searched the PubMed database for literature relevant to constructing a digital maturity evaluation framework and reports about the most prominent international examples of existing evaluation frameworks. The search strategy for locating sources included broad terms such as “evaluate” AND “digital maturity,” as well as more specific terms such as “health information exchange” AND “evaluation.” These specific terms were essential because, in the health service, digital maturity is usually misrepresented and not thought of as a universal advancement, but rather is defined by the individual digital systems or programs that aim to support maturity. Health information exchanges (HIEs), while still only a part of digital maturity, share similar goals with overall maturity in that they aim to mobilize health care information electronically across systems. Therefore, HIE was a useful proxy term for digital maturity in this literature search. We filtered results for relevance to health care and medicine. Finally, we intentionally made the search strategy broad to return papers about all evaluations of digital maturity whether they were specific to one care setting or across care pathways. We searched the PubMed database for literature published between 1995 and 2015 using the following Boolean search strings: (1) “evaluate” AND “digital maturity”, (2) “monitor” AND “digital maturity”, (3) “health information exchange” AND “evaluation”, (4) “health information exchange” AND “monitor.”

### Review Strategy

**Figure 1:**
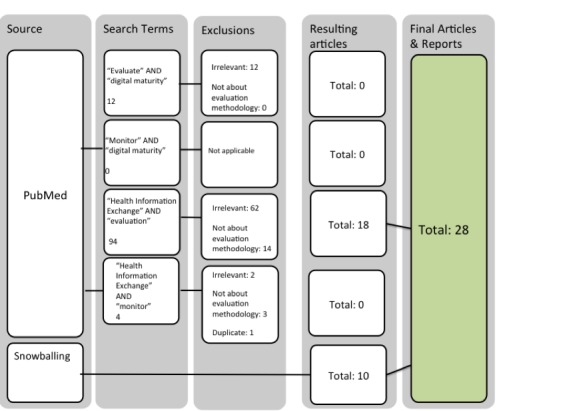
Literature search and review strategy flow chart.

As the flow chart in Figure 1 shows, the search strategy returned over 110 papers, but most were either irrelevant to the goals of the review or did not provide information that contributed to a better understanding of digital maturity evaluation. Inclusion criteria specified that papers must be pertinent to the health service and maintain a focus on the evaluation and some component of digital maturity, such as HIEs. We applied exclusion criteria if papers solely concerned the experience or outcomes associated with digitally mature systems rather than evaluation. Papers were also excluded if they only reported the results of an evaluation and contained no methodological insight. The search returned 18 peer reviewed papers, from which snowballing techniques yielded 7 more peer reviewed papers and 3 relevant reports, totaling 28 included sources.

### Analysis and Framework Development

We analyzed the papers to draw out their contribution to digital system evaluation. This included identifying how to evaluate systems, what to measure, and at what point. It also included a specific examination of how the patient perspective was accounted for within the evaluation. For instance, this included exploring whether the evaluation methodology discussed how to make sure a system was evaluated across the patient pathway or if indicators of success were relevant to patients.

We then used the results from the literature search and review to construct a framework. During the analysis we drew out 5 themes (these themes and associated papers are discussed in the Results section). We translated these themes into the levels of analysis for the evaluation framework and used the indicators within the themes as individual scoring points within each level of analysis (see Framework Development subsection in the Discussion). The resulting framework is situated within a patient-centered paradigm, meaning it measures each of the levels at each major point along the care pathway. This is a departure from existing measurements of digital systems that are rooted in the service-specific goals and are therefore limited in their scope to provoke improvements across the whole care pathway.

## Results

**Figure 2:**
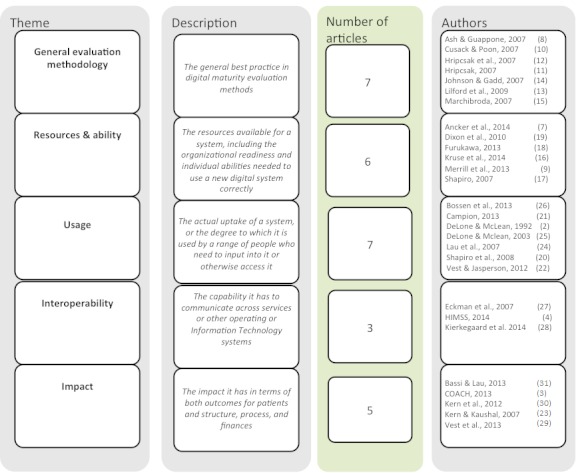
Themes from literature review.

We analyzed findings from the 28 papers identified in the literature search and grouped them into 5 themes to generate the skeleton of a new digital maturity framework: general evaluation methodology, resources and ability, usage, interoperability, and impact. These are detailed in Figure 2.

The literature review confirmed the importance of measuring digital maturity in a way that accounts for its multidimensional nature. The 28 papers reviewed fell into 5 distinct themes, similar to those identified in the background about the concept of digital maturity. The 5 themes—general evaluation methodology, resources and ability, usage, interoperability, and impact—received varying levels of attention in the literature, with the final 2 being the most limited. We discuss these themes, and their associated indicators identified from the literature, in detail to provide context to the development of the evaluation framework. Across all of these themes most papers did not refer to the patient centricity of their approach but rather assumed a service-centric approach, meaning the evaluation considered only one or two settings of care. Although some indicated the importance of certain indicators to patients, any discussion about how the evaluation accounted for success across entire care pathways was distinctly absent.

### General Evaluation Methodology

Many papers emphasized the multifaceted nature of digital maturity specifically for HIEs and the deliberate way evaluations must account for this. Similar to the depictions of digital maturity above, not only do HIEs have many different components, but they also take different forms and have different capacities as they mature [10-13]. Barriers to development, issues around leadership, and commitment are paramount to early-stage evaluations, while stakeholder motivations for engaging with the system are more important as the system advances [11]. As Hripcsak [14] noted,

An HIE project undergoes a series of steps, from early conception to mature maintenance. Reviewing the steps can uncover possible unintended and unexpected effects.

Following a United Hospital Fund (New York, USA) meeting in 2006 to review the best practice for evaluating HIE programs, a series of research projects made inroads into a common evaluation approach and established that their evolutionary nature must be central to appraisals [14]. In other words, using a rigid scoring system or a single metric to evaluate such dynamic and fluid systems is untenable; rather, evaluations must use a comprehensive framework approach [15]. Although a framework should aim to be as objective as possible, considerable evidence suggests that mixed methods add invaluable richness to the data and improve its ability to drive improvement policy [11,16]. Given that assessing how much digital maturity has improved health care quality is very complex, an evaluation framework needs to incorporate nuanced, qualitative feedback from staff and patients [11]. One influential proposal for evaluating digital systems comprehensively and incorporating mixed methods was the “smallball” approach, which disaggregates the components of HIEs and measures them individually across different points in time [17]. Ultimately, digital maturity and the details of operational strategy ought to be closely linked. Just as there can be no single metric for strategy evaluation, findings from the literature explain the futility in narrowing an evaluation framework. A narrow framework would compromise its ability to measure the entirety of digital maturity across the patient pathway in delivering on strategic health system priorities.

In addition to these evaluation strategies, the literature also reveals that HIEs and digital maturity have a wide range of stakeholders to whom each of the components matter differently. Comprehensive evaluation is not just the inclusion of a variety of metrics, but also separate assessments of those metrics as they pertain differently to patients, providers, and policy makers. A framework must span care settings and produce measures from all service levels, as well as the regional and national levels [18].

### Resources and Ability

The studies described above compel the development and use of a multifaceted evaluation framework, and a related body of literature suggests what metrics the framework should incorporate. This research indicates the importance of including readiness measures as a way to assess the extent to which HIEs evolve within an environment conducive to their success. This includes measures of organizations’ existing technology, cultural norms, and leadership to provide context to system functioning and user uptake [10].

More specifically, studies demonstrate the value of gathering information about institutional resources and existing programs that, if insufficient or hostile to the entrance of an HIE, hinder the success of digital systems [19,20]. This refers to finances, staff capacity, experience and willingness, and the existing protocols for information exchange [21]. For instance, inconsistent goals, project rework, and underdeveloped resources are chronic barriers to HIE success, which should be accounted for in the evaluation [12]. Further research explains the importance of quantifying implementation effort when the system is younger, and advancing into usage and then cost metrics only as the system matures [22].

### Usage Measures

Usage can be evaluated in a variety of ways: it can be defined as the volume of information transmitted, the duration and specific activity of users, or simply as the number of login sessions [23,24]. Campion and colleagues [24] used login sessions to explore 3 communities in New York State and the differences in usage among the various stakeholders. They explained that one important measure is whether patient summary data are displayed by default on logging into the system, as this was one of the most significant predictors of patient usage [24].

One of the largest studies about measuring HIE usage demonstrates that usage measurements can be more robust and meaningful when differentiated by activity during use [25]. Vest and Jasperson [25] stratified usage into 5 classifications: minimal usage, repetitive searching, clinical information, mixed information, and demographic information. These types of usage varied by the user’s role. For instance, minimal usage was highest among physicians and clinical information was highest among nurses. This is critical to include in an evaluation framework, as it quantifies how the system is being used and to whom changes would be most impactful [25]. These different types of usage, and their associations with specific roles, help target improvement strategy and evaluate where return on investment can be maximized.

Usability needs to be at the forefront of design, as engendering use is the crux of system development and, without it, indicators at later stages of maturation might be irrelevant [26]. Some evaluation strategies already exist to measure this area. For instance, the information benefits evaluation framework, a nonindustry-specific evaluation model used to evaluate HIEs in Canada, addresses usage and usage type extensively [4,27]. However, while it is appropriate for measuring usage, this framework is not necessarily suitable for measuring digital maturity as a sectorwide advancement for patients, as it neglects organizational, cultural, policy, and other external factors [27]. DeLone and McLean, authors of the parent system, more recently conducted a 10-year review of the system and suggested that a renovated system should include service quality as a new dimension of information system success, which is intended to enhance its applicability throughout the health service [28,29].

### Interoperability

Moving beyond usage, studies have suggested measures for evaluating digital systems’ ability to communicate across settings, as this is central to range and depth of their impact in an integrated, patient-centric health service. In addition to syntactic interoperability, this also includes semantic interoperability. Semantic interoperability, or the harmonization of clinical terminology across care providers, settings, and systems, is particularly important for developing a workable exchange of information [30].

The interoperability of systems for all stakeholders is vital to the systems’ effectiveness in terms of achieving an apparatus for patient centricity; however, research suggests that HIEs are often faulty, with poor ability to communicate across service settings and care sectors [31]. Additionally, evaluating to what extent HIEs are connected across geographic regions is crucial to appraising digital maturity in its entirety, rather than a series of one-off exchanges of information [32].

A health care-specific model for evaluating digital systems is the continuity of care maturity model, which addresses the “convergence of interoperability, information exchange, care coordination, patient engagement and analytics” [6]. It builds on the EMR adoption model, a framework that helps services benchmark their success in using EMRs. This model does move toward a whole systems approach, as it demonstrates the importance of measuring each point along the digital maturity continuum, from establishing electronic systems, to making sure they are interoperable, to evaluating their effectiveness. However, at its core it is designed to be used by individual services to improve their digital functioning, and consequently it has no mechanism for detecting holes in maturity in other services or care settings that might affect overall maturity of the system. This undermines its ability to capture digital maturity holistically and generate improvements that will be relevant across the patient pathway.

### Impact

Moving toward impact, studies suggest that it is necessary to depart from service-specific measures and assess impact across the health care landscape [26]. This can be done by determining how the information structure or other digital program offers public utility [33]. Impact in terms of cost can also be evaluated across stakeholders. Cost metrics focus on the functionalities enabled by HIEs that save money, including HIEs’ ability to generate alerts when there are orders placed for expensive medications or redundant laboratory orders [34]. A systematic review of HIE cost evaluations demonstrates that cost savings were associated with HIE use to a small degree, but there needed to be better, more standardized ways of measuring and reporting cost evaluations [35].

**Figure 3:**
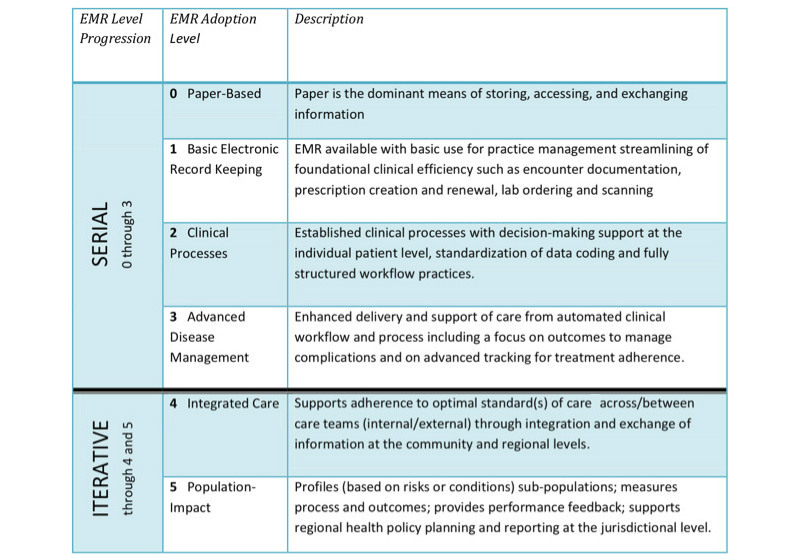
Canadian electronic medical records (EMR) adoption and maturity model. Reproduced with permission from Canada's Health Informatics Association (COACH) [3].

Canada’s Health Informatics Association (COACH), as Figure 3 [5] shows, reviewed 4 impact evaluations of EMR systems. They proposed an EMR-specific evaluation framework that progresses from serial to iterative stages. Although this evaluation model exemplifies measuring a system across a variety of metrics over a period of maturity, it also does not necessarily capture digital maturity as a multiservice or whole systems concept. As a result, it cannot always indicate whether the digital system under investigation is compromised by lags in other care settings.

## Discussion

### Limitations

The remit of PubMed (the database we used to search for papers) includes publications on health care, life sciences, and biomedicine; while it is unlikely that PubMed excluded any relevant sources, we could have augmented the results of the literature review through searching in other databases. More important, relevant sources may exist in harder-to-identify gray literature. Furthermore, although we broadened the search terms to be as inclusive as possible, there is still a bias toward digital maturity for information exchange rather encompassing digital maturity application in all aspects of service delivery

### Analysis

Despite the wealth of information in the literature about how best to evaluate the components of digital maturity, these have not been synthesized into a digital maturity evaluation framework. The first theme, the general evaluation methodology, demonstrates a strong emphasis in the literature on digital maturity being a multidimensional concept. Furthermore, it is clear from the other 4 themes—resources and ability, usage measures, interoperability, and impact—that these must all be captured to evaluate digital maturity holistically. Equipped with the general evaluation methodology and indicators of the 4 levels of maturity, it is necessary to establish at what level they should be measured. The literature also exposes the range of stakeholders who benefit from digital maturity and the growing need in the health service to account for each of the 4 dimensions across different points along the care pathway.

However, our analysis found no evidence in the existing literature of attempts to evaluate digital maturity across the entire care pathway despite the fact that this is necessary to account for all indicators of success. The tendency of existing evaluations, as evidenced by the information benefits framework, the continuity of care maturity model, and the COACH models, is that they place the goals of a specific service at the center of the evaluation, or otherwise fail to capture information about maturity across the whole system. When a framework like this is applied at the service level, or to assess the maturity of a single program within a single service, it risks overlooking the external issues that are most fundamental to the success of a system and overall digital maturity. For instance, these models do not capture where there are resource shortages in one setting or poor interoperability in another. This is critical, as evidence suggests substantial disparities in the quality and use of digital technologies across care settings. A recent survey (Centre for Health Policy, Imperial College London, unpublished data, 2015) demonstrated that IT in secondary care lags the IT systems in primary care, and that secondary care doctors do not have access to all necessary records from primary care. This is not only because primary care records are not interoperable, but also because the digital resources within secondary care are not as advanced.

The evaluation of digital maturity should be able to map the advances of digital systems such that they can facilitate integrated care, better coordination, and improved patient experience across a whole pathway. Measures produced from existing frameworks cannot indicate improvements that would have a sectorwide benefit and be most meaningful to patients who experience care as a pathway, not an individual service. The framework proposed below is designed to measure digital maturity as a driver of integration and improved patient experience. It works from a paradigm with patients at the epicenter, surrounded by their community, primary care, and secondary care to mirror the patient pathway and measure digital maturity across it.

### Framework Development

As opposed to current approaches, this paradigm enables a new way of thinking about the 4 primary areas of digital maturity across a patient pathway. Thinking about the service landscape like this, with the goals and needs of the patient at the center, it becomes possible to envisage new digital solutions that cross the boundaries of traditional service arrangements. Our framework for evaluating systems across all care settings promotes more holistic quality improvement and surpasses frameworks that assess the impact for patients in only one portion of their pathway. We used the 5 themes we identified in the literature review to build a comprehensive framework; we used the general evaluation methodology theme to inform the approach to framework construction, while we translated the other 4 themes into 4 levels of maturity to measure across the patient pathway.

The framework (Multimedia Appendix 1) presents open-ended questions for an evaluator to answer. The open-ended quality is deliberate, as it is meant to provide a standard set of questions but allow for nuances to be captured. The scope for qualitative appraisal is important to measure the evolving progress of digital maturity; however, it is also useful to apply a metric for success. The framework operates in a user-friendly way, in that each question can receive 1 point for a positive answer, meaning each column and row will have a total score. Evaluators can pinpoint areas for improvement by looking at individual negative scores and can identify broader areas that need work through the column and row scores. The overall score out of 58 also provides a comprehensive score for longitudinal benchmarking.

For example, it is possible to analyze mHealth in this framework. mHealth uses phones or tablets to collect patient data including vital signs and general personal health monitoring. mHealth technology can then be used to relay these data to clinicians and community health workers in near real time. Data can also be shared between health settings. If applied well, mHealth technologies have a distinct potential for digital maturity. Such technologies could be evaluated in the home setting to see whether they work for the target population, are easy to use for all patients in the target population, whether they work with the devices patients already have and know how to use, and whether they make a difference without an overwhelming cost or burden. The maturity of the mHealth technologies could then be evaluated at the community level for data transferred to community health workers and the primary and secondary care levels to understand its maturity across the care pathway.

While this framework could be used to evaluate the success of a digital system at 1 service level by isolating 1 row, the overall maturity score is dependent on a sectorwide patient understanding. It highlights where gaps in maturity exist, which presents an opportunity to address the specific shortcomings. This allows subsequent improvement work to be thoroughly patient centric, as it will be intended to support digital maturity across their care pathway. If there is a gap in any box, a solution can be designed that will have a ripple effect and bolster the whole maturity score; this precipitates digital maturity along a patient pathway, so that success in one area is not stymied by failure in another.

### Next Steps

Understanding the utility of this framework will require a trial period of applying it in different health care contexts and comparing scores on certain digital systems. This will help identify whether any areas are missing from the framework. Furthermore, it will be important to gather feedback from evaluators to make sure that the framework is user friendly and well received. Application of the framework has the potential to introduce a standard approach to evaluating digital maturity and mobilize internal benchmarking of digital systems’ maturity.

### Conclusion

The idea that digital maturity is a sectorwide advancement centered on a principal group has been established in other sectors as evidenced by the citizen-centric method of evaluation in e-government. However, in the health sector the progression and success of digital systems has been measured primarily within the confines of individual services’ or care settings’ performance. This does not capture the entirety of digital maturity but, more important, it does not indicate whether a new digital system is capable of helping patients at all points along their pathway.

In order for advancements in digital technologies to proliferate patient benefit in terms of care coordination and enhanced information, digital maturity needs to be conceptualized as a sectorwide, patient-centric measure. Using the literature available on theories behind how to measure the parts of digital maturity, best practice on how to gather indicators of its component parts, and examples of existing evaluation frameworks, our study proposes a contemporary framework that captures 4 key domains of digital maturity across the patient pathway, to pinpoint how digital maturity can be most meaningfully improved.

## Appendix

Multimedia Appendix 1 Digital Maturity Evaluation Framework.

## Abbreviations

COACH: Canada’s Health Informatics Association
EMR: electronic medical record
HIE: health information exchange
IT: information technology
NHS: National Health Service
