# Supplementary material for: A Patient-Centered Framework for Evaluating Digital Maturity of Health Services: A Systematic Review
Source: J Med Internet Res. 2016 Apr 14;18(4):e75. doi: 10.2196/jmir.5047 (PMC4850277; doi:10.2196/jmir.5047)
Supplement: Multimedia Appendix 1 [file jmir_v18i4e75_app1.pdf]

	Capacity/ Resource	Usage	Interoperability	Impact	Total
**Home**	• Does the patient know how to use the part of the digital program that is relevant to them?<br>• Can the patient physically use the part of the digital program that is relevant to them?<br>• Has the technology been tested for relevance with the particular patient population?	• Are patients using the digital program as they are intended to (ie, logging on to it, inputting into it, staying on it for a prolonged length of time)?<br>• Are patient activation scores higher after using it?<br>• Is patient summary data displayed by default in information exchanges?<br>• Is the digital program accessible for people with special needs?	• Is the digital program compatible with the typical devices patients use (ie, can they be used on personal phones, tablets, TV/iTV or computers)?<br>• Can the digital program communicate to/from the home setting to the other three care settings?<br>• Does the digital program use open API to allow future enhancement and adaptation with digital inflation?	• What is the impact to patients in terms of personal health outcomes?<br>• What is the impact to patients in terms of their experience of care? (ie, does it make accessing services and treatments easier? Does it make them feel more involved in their care?)<br>• What is the cost to patients?	Total / 13

**Community**	• Can community service staff use the part of the digital program that is relevant to them?<br>• Can caregivers and family assist patient in using the digital program that is relevant to them?<br>• Do community services have the necessary staff time to use the part of the digital program that is relevant to them?<br>• Do community services have the financial means to have the necessary technology?<br>• What are the organizations existing procedures for information exchange?	• Are community clinicians, social service personnel and caregivers using the digital program as they are intended to?<br>• Have service leaders explained the importance of using the system?<br>• What is the volume of data transmitted from community services?<br>• How long do community service staff and patients stay logged into the system?<br>• What different types of activity are	• Can the digital program communicate with the IT programs already in place in the community services?<br>• Can the digital program communicate to/from the community setting to the other three care settings?	• What is the impact to community services in terms of structure, process, outcomes and finances?<br>• Does the digital program have signaling capabilities to alert community care when costly services are being requested?	• Total 16

**Primary Care**	• Can GPs and staff use the part of the digital program that is relevant to them?<br>• Do GP services have the necessary staff time to use the part of the digital program that is relevant to them?<br>• Do GP services have the financial means to have the necessary technology?<br>• What are the organizations existing procedures for information exchange? Are there any organizational culture issues that might prohibit use of the system?<br>• What are the	• Are GPs using the system?<br>• Have service leaders explained the importance of using the digital program?<br>• What is the volume of data transmitted from GP services?<br>• How long do GP service staff and patients stay logged into the digital program?<br>• What different types of activity are occurring within primary care?	• Can the digital program communicate with the IT programs already in place in the GP services?<br>• Can the digital program communicate to/from the primary care setting to the other three care settings?	• What is the impact to GP services in terms of structure, process, outcomes and finances?<br>• Does the digital program have signaling capabilities to alert primary care when costly services are being requested?	Total / 14

**Secondary Care**	• Can hospital staff use the part of the digital program that is relevant to them?<br>• Do hospitals services have the necessary staff time to use the part of the digital program that is relevant to them?<br>• Do hospitals services have the financial means to have necessary technology?<br>• What are the organizations existing procedures for information exchange?<br>• Are there any organizational culture issues that might prohibit use of the system?<br>• What are the	• Are hospital staff using the digital program?<br>• Have service leaders explained the importance of using the digital program?<br>• What is the volume of data transmitted from acute services?<br>• How long do acute service staff and patients stay logged into the system?<br>• What different types of activity are occurring within hospital care?	• Can the digital program communicate with the IT programs already in place in the hospital services?<br>• Can the digital program communicate to/from the secondary care setting to the other three care settings?	• What is the impact to secondary care services in terms of structure, process, outcomes and finances?<br>• Does the s digital program have signaling capabilities to alert acute care when costly services are being requested?	Total / 15

Total / 21	Total / 19	Total / 9	Total /9	Overall Total / 58
